# Vascular Plant Herbarium at the Kandalaksha Strict Nature Reserve (KAND), Russia

**DOI:** 10.3897/BDJ.8.e59731

**Published:** 2020-12-02

**Authors:** Mikhail N. Kozhin, Alexander N. Sennikov

**Affiliations:** 1 Lomonosov Moscow State University, Moscow, Russia Lomonosov Moscow State University Moscow Russia; 2 Kandalaksha Strict Nature Reserve, Kandalaksha, Russia Kandalaksha Strict Nature Reserve Kandalaksha Russia; 3 Avrorin Polar-Alpine Botanical Garden-Institute, Apatity, Russia Avrorin Polar-Alpine Botanical Garden-Institute Apatity Russia; 4 University of Helsinki, Helsinki, Finland University of Helsinki Helsinki Finland; 5 Komarov Botanical Institute, Saint Petersburg, Russia Komarov Botanical Institute Saint Petersburg Russia

**Keywords:** angiosperms, Barents Sea, databasing, ferns, gymnosperms, Kola Peninsula, Murmansk Region, lycophytes, plant distribution, Republic of Karelia, Russian Lapland, White Sea

## Abstract

**Background:**

The present-day demand for digital availability of distributional data in biodiversity studies requires a special effort in assembling and editing the data otherwise scattered in paper literature and herbarium collections, which can be poorly accessible or little understood to present-day users and especially automatic data processors. Our project on developing the information resource for the vascular plant flora of Murmansk Region, Russia, includes processing and making digitally available all the data on the taxonomy and distribution of this flora. So far, published distribution maps are limited to the old set in the *Flora of Murmansk Region* (published in 1953–1966) and the *Red Data Book of Murmansk Region* (ed. 2, published in 2014). These publications did not take into account the main part of the herbarium collections kept at the Kandalaksha Strict Nature Reserve, which are the basis for numerous local publications that appear scattered and, therefore, little accessible nowadays.

**New information:**

We present a complete dataset of all holdings of vascular plants in the Herbarium of the Kandalaksha Strict Nature Reserve, totalling 10,218 specimens collected during 1947–2019, which are referable to 764 species and 19 subspecies. All specimens were georeferenced with the utmost precision available. This dataset offers a complete and dense coverage of the Nature Reserve's territory (islands and adjacent mainland coastal areas of the Barents and White Seas, Murmansk Region and Republic of Karelia, Russia); these data are little represented in herbarium collections elsewhere.

## Introduction

Nature protection requires complete and up-to-date inventories of the protected object. Inventories are based on vouchers, which support the research and ensure the opportunity for quality control in future studies (Culley 2013). This aim cannot be achieved without access to the background materials, which are typically stored locally and may be disorganised or difficult to interpret.

The Herbarium of the Kandalaksha Strict Nature Reserve (KAND) is a curated scientific collection that is a repository for voucher specimens (mostly vascular plants, but also cyanoprokaryotes, lichens, fungi, algae, mosses and livervorts) collected in the Nature Reserve and its adjacent areas. With its 80-years-long history, the Herbarium contains ca. 16,000 specimens. These holdings place the Herbarium fourth amongst the botanical collections of Russian nature reserves and 85^th^ among all registered Russian collections (according to Index Herbariorum, http://sweetgum.nybg.org/science/ih/).

Currently, the KAND herbarium is one of the key botanical collections from Murmansk Region of Russia. Despite its importance, the collection is little accessible due to its remote location in Kandalaksha, a small town in northern Russia, far away from major botanical centres with taxonomic activities (like Komarov Botanical Institute or Moscow State University). Another factor hindering the use of its collection was the former lack of curation and, consequently, the insufficient level of accessioning of older collections.

When the collections became completely and properly organised, it was considered timely to include the KAND Herbarium into global information systems, in order to make it accessible to the public and involved in modern biodiversity research projects. The present contribution aims at bringing the distributional data accumulated in the Nature Reserve to the public; besides, it complements the distributional dataset derived from the *Flora of Murmansk Region* (Gorodkov 1953, Poyarkova 1954, Poyarkova 1956, Poyarkova 1959, Poyarkova 1966), which was published elsewhere (Kozhin et al. 2020a, Kozhin et al. 2020b).

## General description

### Purpose

The present article aimed at digital representation and making publicly available the data on the distribution of vascular plants from the herbarium collection of the Kandalaksha Strict Nature Reserve.

### Additional information

**Origin of collections**

The Kandalaksha Strict Nature Reserve was founded in 1932 as a legally-protected area and a research institution aimed at monitoring and studying animals and plants of that area (Karpovich 1988). To document the plant diversity of the Nature Reserve, its herbarium collection was established in line with the practice of biodiversity studies in the Union of Soviet Socialist Republics (USSR).

The original purpose of the Nature Reserve was the protection and study of the common eider, *Somateria mollissima*, to protect its nesting places and to regulate the exploitation of its valuable down (Dubrovskii 1936). Plant research and protection was developed at a later stage, although some examination of the vegetation cover was included already in the background work by A.N. Dubrovsky (Dubrovskii 1936), who explored the territory and drafted the prospect of the future Nature Reserve in an expedition directed by the Leningrad Forestry Research Institute. Although the expedition intended to preserve botanical collections (Dubrovskii 1934), no herbarium specimens seem to have survived from those times. The pilot studies of plant communities in the Nature Reserve by T.P. Nekrasova in June of 1941 were interrupted by the war and brought no botanical collections either (Breslina 1968, Bianki 1998).

The first botanical collection for the Nature Reserve was made during the dissertation study by N.S. Parfentieva, who started mass gathering of dried plants during her employment in 1949–1950 and continued sporadically in 1958 and 1959 (Vorobieva 1987). Her work covered the territories along the Barents Sea coast and yielded a collection of over 1,500 specimens (Karpovich 1967). This work was also interrupted and remained unfinished; the collections were divided and deposited at KAND, MW (specimens from 1958) and KPABG.

In 1957 and 1959, a similar study was performed on the islands along the southern coast of the White Sea by G.M. Sinkova (Breslina 1968, Vorobieva 1987), who was a PhD student based at KPABG. She collected ca. 2,500 specimens of vascular plants, which were largely deposited at LE and KPABG, of which only a very small amount (107 specimens) arrived to KAND. This study was also left unfinished.

Minor botanical collections were made by zoologists employed at the Nature Reserve. For example, T.V. Koshkina collected some specimens during 1952–1956, which were amongst the early collections from the White and Barents Seas (Vorobieva 1987).

An important source of collections was student summer camps organised by the Department of Vascular Plants of the Moscow State University since the 1960s. In those years, the student expeditions, led by V.N. Vekhov (assistant professor) and N.E. Bogdanova (technical assistant), explored the Kem-Ludy Archipelago in 1962 (Bogdanova and Vekhov 1969a) and the Velikii Island in 1963–1964 (Bogdanova and Vekhov 1969b). The resulting herbarium collections were deposited partly at KAND (775 specimens), but largely at MW, with duplicates distributed from MW on an exchange basis.

The greatest contribution to the collections and the study of vascular plants of the Nature Reserve was made by I.P. Breslina. She performed a study of the flora and vegetation of the Kandalaksha Skerries of the White Sea in 1963–1966 (Breslina 1968), those of the Sem Ostrovov Archipelago of the Barents Sea in 1964–1967 (Breslina 1967, Breslina 1969) and in the Ainov Islands of the Barents Sea in 1968 (Parfentieva and Breslina 1969). Breslina regularly collected hundreds of specimens per year, which were rapidly identified and retained the highest quality of preservation. Taxonomically most difficult parts of her collections were identified by recognised experts: T.V. Egorova and N.N. Tzvelev (LE), R.N. Schljakov and O.I. Kuzeneva (KPABG). Some parts of her collections were donated to LE and KPABG and, therefore, were included in taxonomic treatments published in the *Flora of Murmansk Region* (Gorodkov 1953, Poyarkova 1954, Poyarkova 1956, Poyarkova 1959, Poyarkova 1966), although the particular herbarium specimens kept at KAND had not been examined and used for this publication.

After Breslina had been fired from her position of scientist at the Nature Reserve at the end of the 1960s, she took the position of forest ranger for the Kem-Ludy Archipelago, but her collecting activity was interrupted. In 1976, Breslina accepted a scientific position at KPABG where she continued her botanical studies on the islands of the White Sea. In 1977–1978 and 1984–1987, Breslina extensively studied the islands in the Porya Bay (Breslina 1987), which were added to the Nature Reserve in 1977. Her herbarium specimens from the later period were deposited at KPABG.

Breslina’s successor, V.A. Tsarkova (Vorobieva 1987) was not active in botanical collections. During Tsarkova’s term, in 1971–1974, only some 20 specimens were added to the herbarium holdings.

The territory of the Nature Reserve was continuously expanding to include new patches of protected land. Floristic inventories were required to cover the newly-acquired territories. This work was performed by E.G. Vorobieva, who was employed as a botanist during 1975–1994. Between 1976 and 1989, she studied the flora of the Luvenga, Olenii (Vorobieva 1996b), Kibrinskii (Vorobieva 1986), Tarasikha (Vorobieva 1996a) and Vachev (Vorobieva 1989) archipelagos and updated the information collected by Breslina from the Severnyi Archipelago (Vorobieva 1986). Many students from Leningrad (T. Gorokhova, I. Turevskaya, L.A. Diulina, E.I. Nikitina, S.A. Novozhilova and N.G. Khrenova), Kazan (M.V. Bubnova and V.G. Lavrova), Gorkii (L. Kuznetsova) and Pskov (V. Stalnova) assisted in this work. Vorobieva and her students made extensive herbarium collections, which remained untreated for a long time.

In the same period of time, the Nature Reserve employed another botanist, A.B. Georgievskii, who mostly studied plant communities and vegetation. In 1977, he explored the Gavrilov Islands (Georgievskii 1981) with a team of students and assistants (L.S. Georgievskaya, L.V. Kuznetsova, V.Yu. Neshataev and E.G. Redneva). Similarly, during 1977–1982, he mapped the vegetation of the Kovda Peninsula and the Porya Bay (Vorobieva 1987). These studies were documented with very few herbarium specimens.

Between 1990 and the beginning of the 2000s, the Nature Reserve was in a period of scientific inactivity due to limited funding. During this period, only minor botanical collections originated from the vicinities of Dalnie Zelentsy and the Gavrilov Archipelago by T.D. Paneva, M.Yu. Kupryukhina, D.M. Gerasimov and A.D. Vital (Paneva et al. 2006).

There was a long-term exploration and monitoring of the flora and vegetation of the Kem-Ludy Islands made by pupils and teachers of the Moscow Grammar School 1543, as well as students of the Moscow State University, which took place during 2001–2017 (Shipunov and Abramova 2006). Since 2002, student summer camps were organised by the Department of Vascular Plants of the Moscow State University by D.D. Sokolov. The resulting collections were deposited at MW with only a few specimens at KAND.

In the 2000s, N.G. Panarina (Khrenova) continued her studies on the flora of the islands of the Kandalaksha Bay, which were started in the 1980s and culminated with her PhD degree (Panarina and Papchenkov 2005). A number of school pupils from Umba Village (M.N. Kozhin, N.V. Nesterova, T.A. Balaganova, E.A. Osipova and Yu.G. Khrenova) participated in this work. The herbarium material, documenting these studies, was largely deposited at IBIW and only ca. 100 specimens remained at KAND.

In the 2000s, new botanists were employed at the Nature Reserve. During 2002–2004, V.N. Zherikhina (Plyusnina) studied the islands of the Severnyi Archipelago (Zherikhina and Moskvicheva 2006), the Kovda Peninsula and Velikii Island and collected ca. 150 specimens. During 2005–2008, E.N. Sidneva (Svyatova), assisted by E.A. Zakharova, studied seashore meadows of Velikii Island and the Kovda Peninsula and the flora of the smaller islands of the Babie More Bay. They collected a few hundred herbarium specimens, but only 89 specimens were accessioned at KAND.

Since 2008, M.N. Kozhin took the position of botanist at the Nature Reserve and the collection work has been considerably intensified. He studied the flora and vegetation in all parts of the White Sea area in the Nature Reserve. During these works, in 2006 and 2008, he focused on the flora of the Turii Mys with the assistance of students of the Faculty of Geography of the Moscow State University (T.S. Grevizirskaya and N.A. Zhorov). Targeted collections of sedges (*Carex* sp.) were made in 2007 in the Kandalaksha Bay. The most active floristic exploration took place in the Porya Bay during 2009–2014 (Kozhin 2011, Kozhin 2014, Kozhin 2016), with participation of students from the Faculty of Biololgy of the Department of Plant Ecology (T.V. Krutenko and K.B. Popova), the Department of Higher Plants (N.A. Vislobokov) and the Faculty of Geography of the Department of Biogeography (S.V. Dudov, A.P. Ivanov and T.S. Grevizirskaya) of the Moscow State University, Faculty of Psychology of the Saint-Petersburg State University (N.V. Nesterova), and a schoolteacher of biology from Kandalaksha (T.S. Vorobieva).

At the same period, in 2011–2013, E.O. Golovina (Komarov Botanical Institute) studied plant communities on the islands of the Porya Bay and the Srednie Ludy Islands. In 2017, M.N. Kozhin organised an expedition to the islands of the Olenii, Severnyi and Kibrinskii archipelagos and to Molochnitsa Island near the Kovda Peninsula, with participation of E.O. Golovina, S.A. Kutenkov (Institute of Biology, Petrozavodsk) and D.A. Zakharchenko (student of the Department of Plant Ecology, Moscow State University).

In the latest decade, students from the Moscow State University actively and regularly visited the Nature Reserve. In 2009 and 2010, E.A. Gryaznova collected nearly 150 specimens in the vicinity of Luvenga. Since 2012, a student summer camp has been organised by M.N. Kozhin in a few areas of the Kandalaksha Bay. Some students, supervised by Kozhin, based their work at the Nature Reserve: E.I. Vuzman worked on the islands of the Luvenga Archipelago in 2016–2019 (Vuzman and Kozhin 2019) and E.V. Kudr (with E.I. Medvedeva in 2017) studied the vegetation on the islands of the Porya Bay in 2017-2018 (Kudr and Kozhin 2019).

Altogether, ca. 4,000 herbarium specimens have been collected since 2008. These collections were deposited at KAND (ca. 1,500 specimens), MW, H and KPABG.

**History of collection**

The herbarium collection, as a separate scientific item, was established by N.S. Parfentieva in 1949 (Vorobieva 1987). It was not until the end of the 1950s when the collections have been organised and properly identified. In the 1960s, I.P. Breslina started scientific curation of the herbarium collections, which included vascular plants, lichens and mosses. Vascular plant collections were organised taxonomically, according to the *Flora of Murmansk Region* (Gorodkov 1953, Poyarkova 1954, Poyarkova 1956, Poyarkova 1959, Poyarkova 1966) and geographically as two subsets, the White Sea and the Barents Sea. In those times (Breslina 1967, Breslina 1968), the collections included 1459 specimens originating from the White Sea (with 644 specimens from the Severnyi Archipelago) and 975 specimens originating from the Barents Sea (with 859 specimens from the Sem Ostrovov Archipelago). The total number of specimens reached 2434 by the end of the 1960s.

During the next decades, despite the high intensity of inventories in the Nature Reserve, the accessions did not grow actively, because the new collections were largely left unorganised. By 1983, the number of specimens included in the collections was 2651 only (Karpovich 1988), which included 1543 specimens from the White Sea and 1108 specimens from the Barents Sea. In the 1990s, the collections were not curated at all and the later inventory (Moskvicheva 2004) counted the same 2651 specimens.

**Figure 1.**
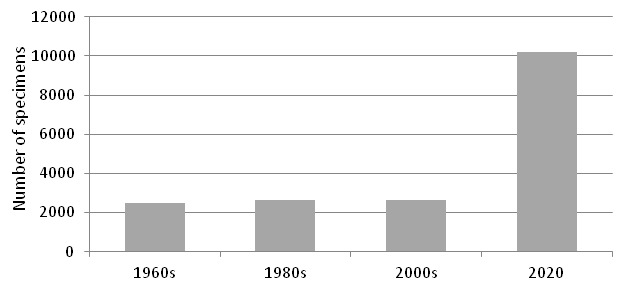
The number of herbarium specimens of vascular plants in the Kandalaksha Strict Nature Reserve according to the annual accessions.

During the 2000s, herbarium curation in the Nature Reserve was resumed due to the active supervision of A.S. Koryakin, vice-director of the Reserve (Goryashko 2014). V.N. Zherikhina and E.N. Sidneva accessioned their own specimens. Since 2006, M.N. Kozhin took the job to identify and organise the collections. At that time, the subdivision of the Herbarium into two subsets was abandoned, but the collections were re-arranged according to the more detailed subdivision of the territory within each species. Now, the collection is organised according to the system of families and genera adopted in Dalla Torre and Harms (Dalla Torre and Harms 1900, Dalla Torre and Harms 1901, Dalla Torre and Harms 1903, Dalla Torre and Harms 1904, Dalla Torre and Harms 1905, Dalla Torre and Harms 1906, Dalla Torre and Harms 1907); species within genera are arranged alphabetically. New additions to the collection were annually accessioned on a regular basis. At present, the herbarium collection of the Nature Reserve numbers 10,218 specimens, which is a four-fold increase compared to the first inventory (Fig. 1). Nevertheless, in the latest years, the accessioning became irregular again, due to the lack of human resources for technical curation of the collections.

**Databasing of the herbarium collection**

The first attempt to make a catalogue of herbarium collections in the Nature Reserve dates back to the 1960s. The information was recorded on paper cards, including species name, locality, habitat, collection date and number of duplicates. This work embraced the specimens collected from Velikii Island and the Severnyi Archipelago, but other parts of the collection were left unfinished. Besides, there were attempts to catalogue specimens collected by certain expeditions: 116 herbarium specimens collected by N.V. Koshkina in Ponoi were catalogued but not preserved, perhaps because of being extraterritorial and thus irrelevant to the Nature Reserve.

A new, complete catalogue on paper cards was compiled by E.G. Vorobieva in 1975–1976, which included species names and localities. This catalogue has never been updated.

**Figure 2.**
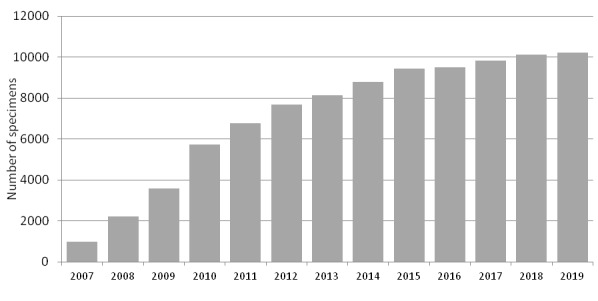
Progress in databasing of the herbarium of vascular plants of the Kandalaksha Strict Nature Reserve.

Since the mid-1990s, the Nature Reserve prioritised electronic databasing of all the vast information accumulated in the Nature Reserve. The databasing of the herbarium of vascular plants has been in effect since 2007, when M.N. Kozhin started to record the information from the publicly-accessible collection and to organise its previously unaccessioned and newly arrived parts (Fig. 2). At present, all historical specimens are completely organised and included in the main collection.

## Project description

### Title

Vascular Plant Herbarium at the Kandalaksha Strict Nature Reserve (KAND), Russia

### Personnel

Mikhail N. Kozhin (project leader, collection identifier, data collector, data manager).

Alexander N. Sennikov (collection identifier, data collector, data manager).

Andrey V. Matveev (developer, web-designer).

### Study area description

**Figure 3.**
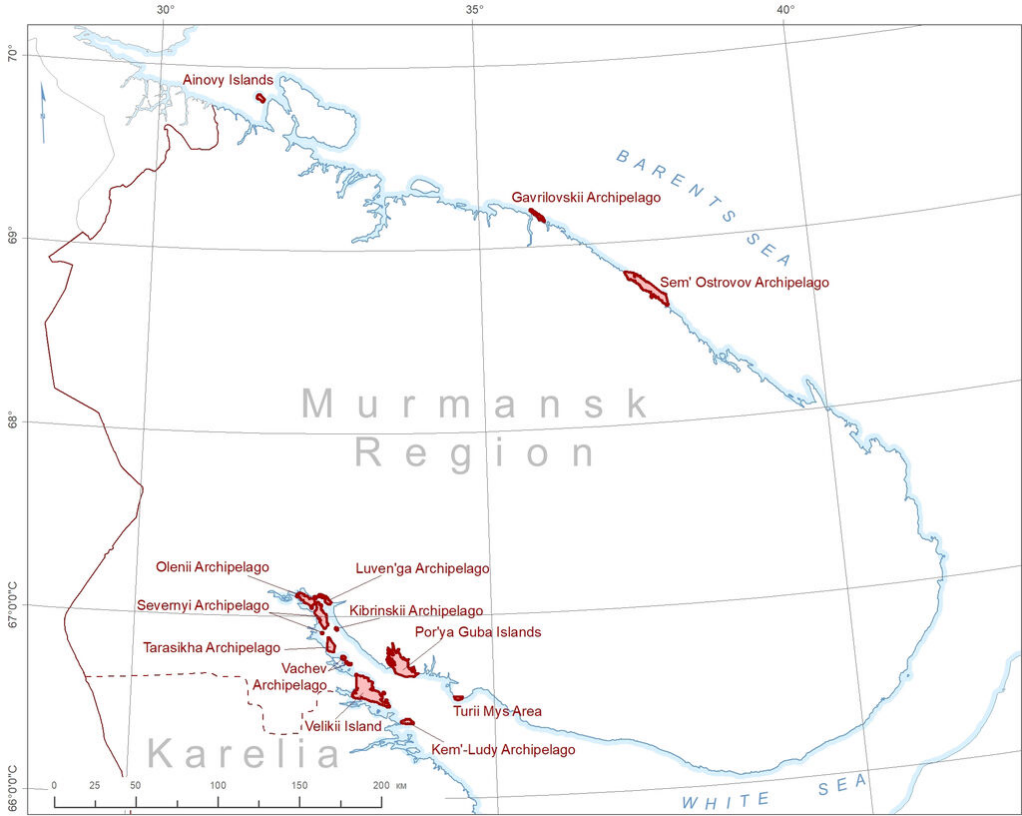
Protected areas included in the Kandalaksha Strict Nature Reserve (Murmansk Region and north Karelia, Russia).

Kandalaksha Strict Nature Reserve is located in the northwest of European Russia and consists of 13 separate areas (Fig. 3), scattered along the sea periphery of Murmansk Region from the border with Norway in the Barents Sea to the Karelian Republic in the White Sea. The Nature Reserve is situated in five administrative districts (Pechenga, Kola, Lovozero, Terskii, Kandalaksha) of Murmansk Region and Louhi District of the Republic of Karelia. The majority of these protected areas are sea archipelagos with adjacent water areas, but the Nature Reserve also includes some coastal mainland areas. Its area totals 705.3 km^2^, of which 70% is sea waters. From its inception, the Nature Reserve was intended for protection of seabirds and sea mammals whose colonies use limited-size coastal areas. As a result, the Nature Reserve is a network of comparatively small areas, separated by the distances of hundreds of kilometres. In 1975, the Kandalaksha Bay of the White Sea, where most of the Nature Reserve’s archipelagos are located, was included in the list of Wetlands of International Importance as a habitat for water birds.

This territory lies almost completely north of the Arctic Circle and its climate is mostly subarctic with a minor influence of the polar climate along the northern coast and in the northern islands (Peel et al. 2007). The yearly mean air temperature is about 0°, the length of the frost-free period is 90–110 days. Due to their proximity to the Gulf Stream, the protected aquatic areas in the Barents Sea are ice-free in winters, but in most severe winters, shallow coastal waters can freeze. The climate in the White Sea is more continental. The territory is divided between two biogeographic regions, arctic and boreal (*Cervellini et al. 2020*). The tundra vegetation is typical for the Barents Sea areas of the Nature Reserve and occurs in a narrow belt influenced by the arctic climate, as dwarf-shrub and dwarf-shrub and lichen communities, whereas islands in the White Sea are covered by the northern taiga zone, represented by pine and spruce-dominated forests (*Gribova et al. 1980*).

### Design description

The current project aims at digital representation and publication of the data on the distribution of vascular plants as represented in the Herbarium of the Kandalaksha Strict Nature Reserve. Data from herbarium specimens were captured into the database and complemented with georeferences (Suppl. material 1). The resulting information was deposited in the database ‘Flora of Russian Lapland’ (https://laplandflora.ru/) and visualised through GBIF (Kozhin and Sennikov 2020a).

The Nature Reserve offers a good representation of the territory of Murmansk Region, covering a number of areas situated in various parts of the territory, with emphasis on coastal areas. Sampling density and coverage in these areas is high and the covered territory has a special value in nature protection and monitoring. Most of the databased specimens are unique, i.e. not represented by duplicates in other herbarium collections.

## Sampling methods

### Sampling description

The dataset has been prepared in the course of a complete inventory of herbarium collections of vascular plants of the Kandalaksha Strict Nature Reserve. The information on the specimen identity and the history of identifications, locality, collection date and collector was captured from mostly handwritten herbarium labels and recorded into a spreadsheet of MS Excel. When in doubt, localities, collection dates and collector names were verified against historical documents kept in the archive of the Nature Reserve.

Specimens were georeferenced using Google Maps or Yandex Maps. Local toponyms were traced using printed large scale topographic and marine maps, Hydrographical description of the White Sea, forest inventory maps for 1976–1977 and 2016 and records in the archive of the Nature Reserve. Georeferencing generally followed the Georeferencing Quick Reference Guide (Zermoglio et al. 2020). The recorded coordinates correspond to the presumed centre of the georeferenced locality. Depending on the locality’s size, the estimated accuracy varied between 5 m and 16.2 km.

The dataset was incorporated into the database of the project ‘Flora of Russian Lapland’ (https://laplandflora.ru/), which is maintained at the Moscow State University.

### Quality control

The original identification, when available, was verified for each specimen. Recognised taxonomic experts were involved in difficult cases: Yu.A. Alexeev, K.P. Glazunova, V.S. Novikov (Moscow State University), A.A. Bobrov (Institute for Biology of Inland Waters, Borok), P.G. Efimov and N.N. Tzvelev (Komarov Botanical Institute). Identifications in apomictic groups (*Hieracium*, *Ranunculus auricomus* s.l., *Taraxacum*) remain incomplete.

## Geographic coverage

### Description

The Herbarium of the Kandalaksha Strict Nature Reserve possesses specimens collected in Murmansk Region and northern Karelia, Russia. The greatest amount of specimens (84.25%) originated from the territory of the Nature Reserve.

The land size of the Nature Reserve totals 20,947 ha. This figure has never been stable; during its history, the territory of the Nature Reserve fluctuated significantly, mostly with expansion by gradual inclusion of new areas or some extra land (islands or mainland coast) adjacent to the existing protected areas (Koryakin 2006).

The territory of the Nature Reserve is subdivided into four forest districts ('lesnichestvo' in Russian), of which three are situated on the White Sea and one is located on the Barents Sea. Herbarium collections are organised according to these forest districts (Table 1).

**Figure 4.**
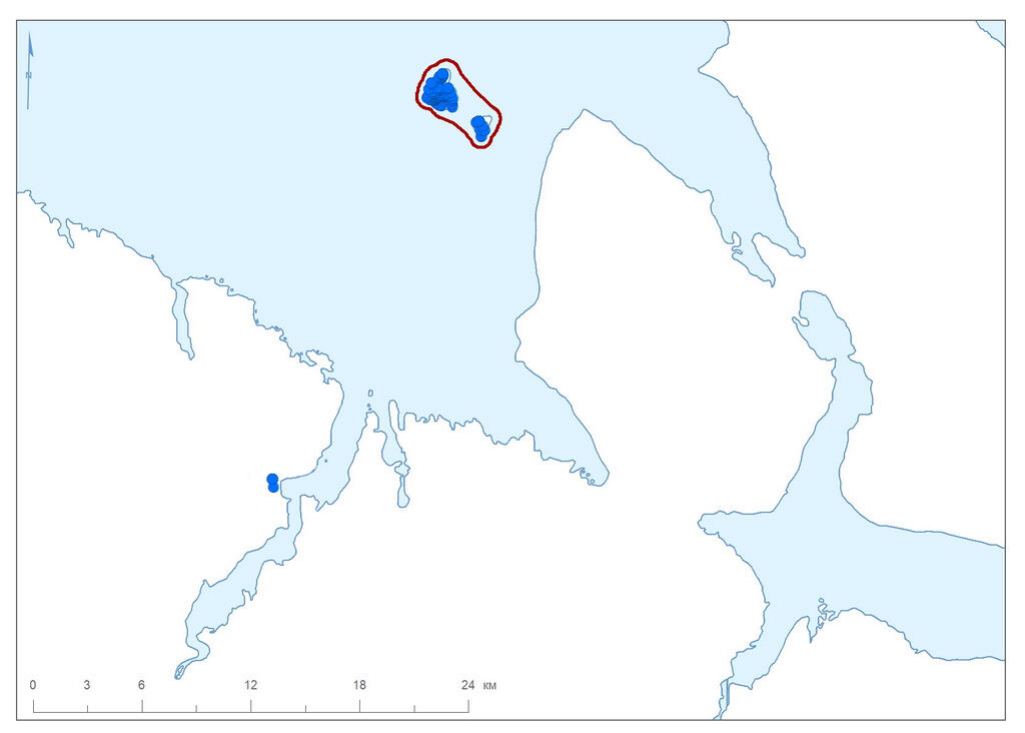
Summary map of all records in the Ainovy Islands and adjacent area, the Barents Sea. Red line denotes a protected area.

**Figure 5.**
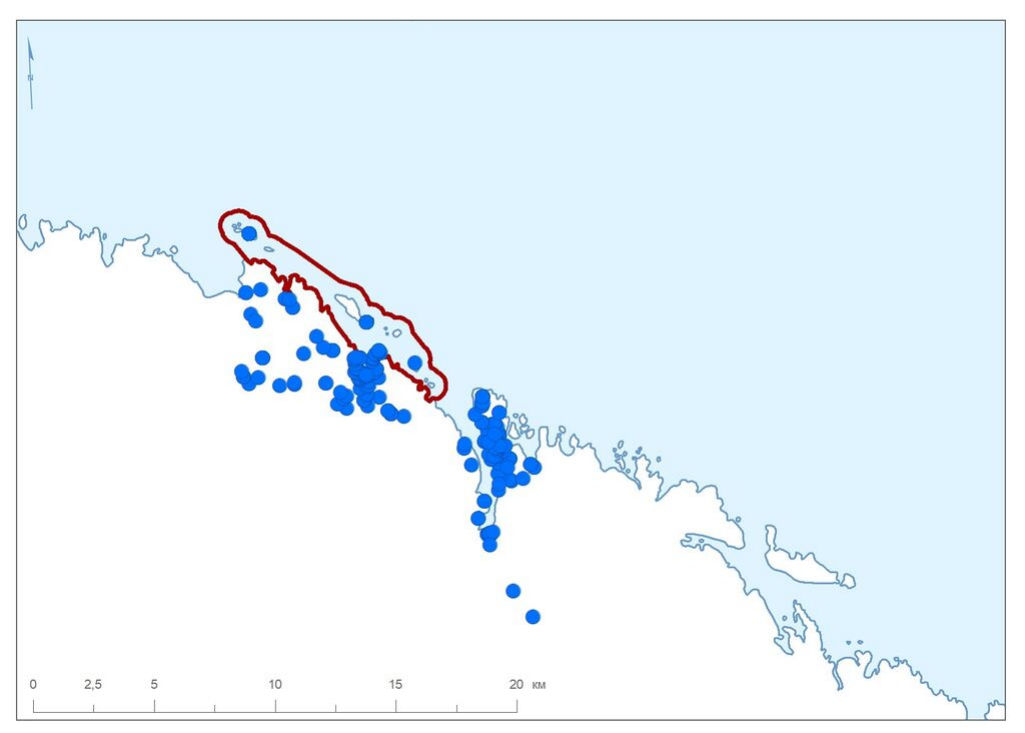
Summary map of all records in the Gavrilov Archipelago and adjacent area, the Barents Sea. Red line denotes a protected area.

**Figure 6.**
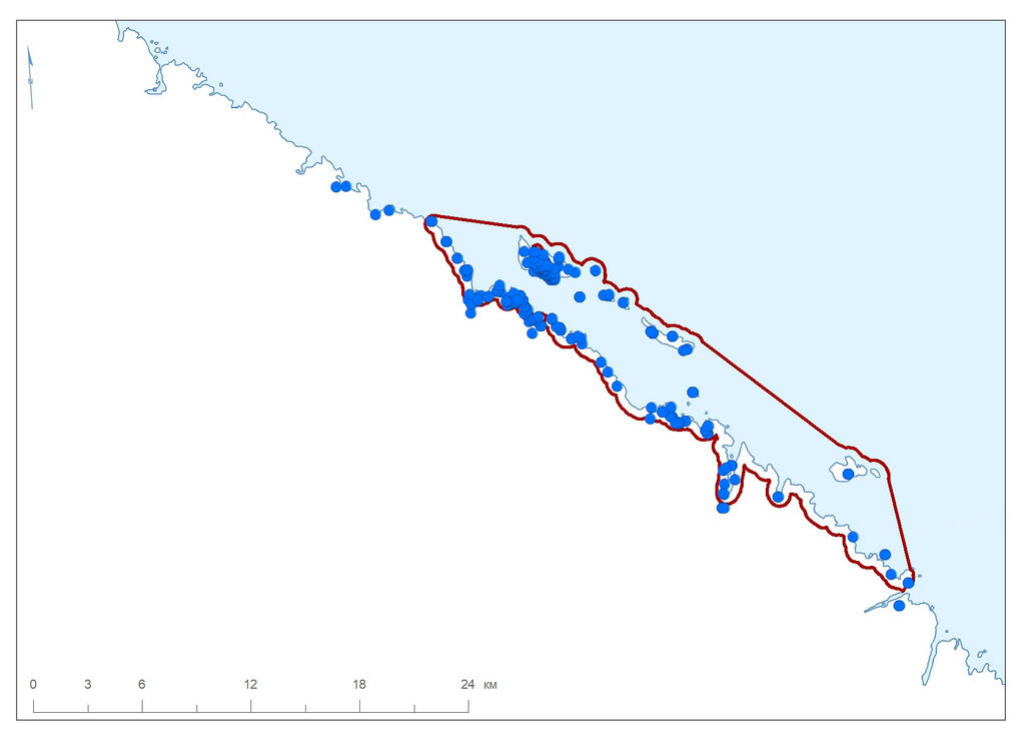
Summary map of all records in the Sem Ostrovov Archipelago and adjacent area, the Barents Sea. Red line denotes a protected area.

The Barents Sea Forest District is made up of three areas. The Ainovy Islands (Fig. 4) are a group of two small and flat islands situated close to the border with Norway. The Gavrilov Archipelago is located in the middle of the Murman Coast; it is poorly sampled because of its small territory and shorter history of strict protection (Fig. 5). The Sem Ostrovov Archipelago is the easternmost area of the Nature Reserve, the largest and the best sampled in the north (Fig. 6, Table 2). Originally (since 1937), this protected area was limited to the islands, but subsequently (1947) expanded to include the adjacent mainland coast.

**Figure 7.**
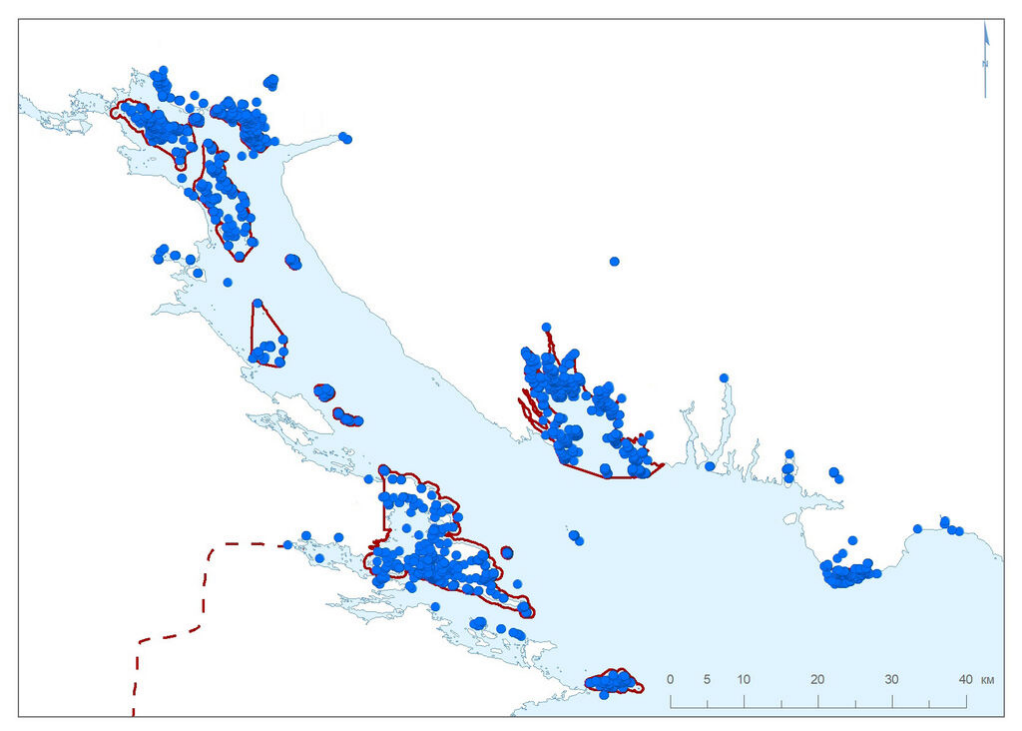
Summary map of all records in the Kandalaksha Bay, the White Sea. Red line denotes a protected area.

In the White Sea, the greatest number of specimens originated from the Severnoe Forest District (Fig. 7, Table 1). The Severnyi Archipelago was the first area protected in the Nature Reserve. It harbours the main research station of the Nature Reserve situated on Ryazhkov Island. Botanical studies at this station started in 1949; this long period of research accounts for the greatest number of specimens accumulated. The Olenii Archipelago, Luvenga Archipelago and Kibrinskii Archipelago are part of this area but protected later and lacking research stations; consequently, the number of specimens collected from those islands is much smaller (Table 2).

The Velikii Island Forest District is situated south of the Severnoe Forest District (Table 1). Velikii Island has the best coverage with herbarium material (1250 specimens), which is explained by the island's large size and long history of protection (since 1947) and the former activity of research stations. The territories neighbouring to Velikii Island, the Kovda Peninsula (mainland) and some smaller adjacent islands, were included in the Nature Reserve later (1967) and, therefore, explored in a less detail. The Vachev and Tarasikha archipelagos are poorly sampled because of the low level of botanical activities (Table 2).

Kem-Ludy Archipelago (protected since 1957), a southern extension of the Velikii Island Forest District, is the only part of the Nature Reserve situated in northern Karelia. This territory is well sampled, but most of the relevant herbarium specimens are kept at MW due to the botanical research conducted by students and teachers of the Moscow State University.

The Terskoe Forest District is situated eastof the Severnoe Forest District and includes two separate areas, Porya Bay and Turii Mys. At present, the territory of the Porya Bay is best sampled with herbarium specimens in the Nature Reserve (Table 2), partly due to a research station situated in Gorelyi Island. Its original territory (since 1967) embraced islands situated in the sea and was expanded in 1977 to incorporate all islands and waters of the Bay. The second area of this Forest District, Turii Mys, has received much attention from botanists since the 19^th^ century (Sennikov and Kozhin 2018, Kozhin and Sennikov 2020b) because of a number of extraordinary plant species restricted to the area. Despite its high importance, this territory was protected only in 1977; most of the specimens originating from the Turii Mys, including historical collections, are kept outside KAND, at H, KPABG and LE.

A significant amount of herbarium specimens at KAND (15.75%) was collected from the territories immediately adjacent to strictly protected areas. Many of such specimens were collected in the vicinity of Dalnie Zelentsy near the limits of the Barents Sea Forest District (Table 1), where an active research station of the Nature Reserve is situated. This territory is planned for inclusion in the Nature Reserve. Many specimens were collected in the vicinity of Luvenga near the Severnoe Forest District, which has been used for student summer camps of the Faculty of Biology of the Moscow State University since 2015, and in the vicinities of Kandalaksha, where the headquarters of the Nature Reserve are situated. Near the borders of the Terskoe Forest District, the extraterritorial specimens were collected mostly from the vicinities of Porya Guba abandoned village; this territory was explored by Breslina in the 1970s and 1980s and is used for the student summer camps nowadays.

### Coordinates

66.41 and 69.84 Latitude; 31.55 and 37.81 Longitude.

## Taxonomic coverage

### Description

The dataset covers all taxonomic groups traditionally treated as vascular plants, i.e. Lycopodiophyta, Pteridophyta (incl. Pteridopsida and Equisetopsida) and spermatophytes (incl. Magnoliophyta and Pinophyta), which were collected and stored at the Herbarium of the Kandalaksha Strict Nature Reserve (KAND). Altogether the collection is represented by 80 families, 307 genera, 764 species and 19 subspecies of vascular plants. The most species-rich families are Asteraceae (82), Poaceae (73), Cyperaceae (70), Rosaceae (51) and Caryophyllaceae (38).

In the herbarium collections, plant names are accepted according to Czerepanov (1995).

## Temporal coverage

**Data range:** 1949-5-30 – 2019-9-07.

### Notes

**Figure 8.**
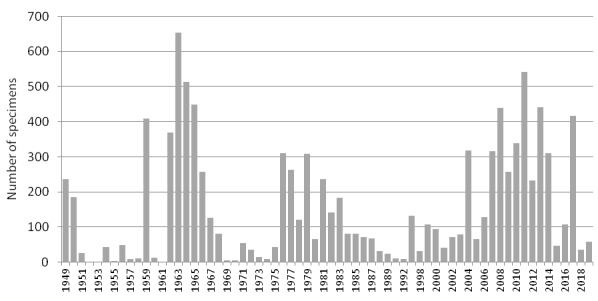
Annual increase in herbarium accessions of the Kandalaksha Strict Nature Reserve.

The dynamics of collecting activities in the Nature Reserve has been highly uneven (Fig. 8). During the 1940s and 1950s, herbarium specimens were collected in a few years only, largely because of logistic problems (Goryashko 2020) and the temporary employments of botanists. The most fruitful period of collecting activity occurred in the 1960s and was linked with the work of Breslina and the expedition of the Moscow State University led by V.N. Vekhov. The following period of depression from the end of the 1960s to 1975 was linked with acute conflicts amongst the scientific personnel. During 1975–1983, the collections annually increased due to the work of E.G. Vorobieva, A.B. Georgievskii and visiting students. Another period of depression (1984–2003) can be explained by political and financial difficulties in the country. During that period, small collections were donated mostly by visiting scientists. Since 2004, the herbarium had been actively increasing with works of V.N. Zherikhina, E.N. Sidneva and M.N. Kozhin. Since 2015, the collecting activities have been casual, with a single significant expedition in 2017.

## Collection data

### Collection name

Herbarium of Kandalaksha Strict Nature Reserve

### Collection identifier

KAND

### Specimen preservation method

Dried specimens of vascular plants are mounted on sheets of drawing paper by sewing and gluing by gummed strips of adhesive paper. Each specimen is labelled and numbered with a running number that is used for databasing. Specimens are inserted in folders and stored in wooden cabinets.

### Curatorial unit

Herbarium specimens are curated as a single item. Specimens within a certain species are further organised according to territories, with the following codes in current use. 0. Locality unknown. Strictly protected areas (green labels): 1. Severnoe Forest District, 2. Velikii Island Forest District, 3. Terskoe Forest District, 4. Barents Sea Forest District. Adjacent areas (yellow labels): 1a. Adjacent area of Severnoe Forest District, 2a. Adjacent area of Velikii Island Forest District, 3a. Adjacent area of Terskoe Forest District, 4a. Adjacent area of Barents Sea Forest District.

## Usage licence

### Usage licence

Other

### IP rights notes

Creative Commons Attribution (CC-BY) 4.0 License

## Data resources

### Data package title

Vascular plants in the Herbarium of the Kandalaksha Strict Nature Reserve (KAND)

### Resource link

https://doi.org/10.15468/vebcs3

### Number of data sets

1

### Data set 1.

#### Data set name

Vascular plants in the Herbarium of the Kandalaksha Strict Nature Reserve (KAND)

#### Number of columns

33

#### 

**Data set 1.** 

Column label	Column description
occurrenceID	An identifier for the Occurrence (as opposed to a particular digital record of the occurrence). In the absence of a persistent global unique identifier, construct one from a combination of identifiers in the record that will most closely make the occurrenceID globally unique
institutionCode	The name (or acronym) in use by the institution having custody of the object(s) or information referred to in the record [KAND]
catalogNumber	An identifier (preferably unique) for the record within the dataset or collection
basisOfRecord	The specific nature of the data record [PreservedSpecimen = herbarium specimen]
recordNumber	An identifier given to the Occurrence at the time it was recorded. Often serves as a link between field notes and an Occurrence record, such as a specimen collector's number
scientificName	The full scientific name, with authorship and date information if known. When forming part of an Identification, this should be the name in lowest level taxonomic rank that can be determined. This term should not contain identification qualifications, which should instead be supplied in the IdentificationQualifier term
taxonRank	The taxonomic rank of the most specific name in the scientificName
family	The full scientific name of the family in which the taxon is classified
genus	The full scientific name of the genus in which the taxon is classified
specificEpithet	The name of the first or species epithet of the scientificName
infraspecificEpithet	The name of the lowest or terminal infraspecific epithet of the scientificName, excluding any rank designation
scientificNameAuthorship	The authorship information for the scientificName formatted according to the conventions of the applicable nomenclaturalCode
taxonRemarks	Comments or notes about the taxon or name
countryCode	The standard code for the country in which the Location occurs [RU]
stateProvince	The full, unabbreviated name of the next smaller administrative region than stateProvince (county, shire, department etc.) in which the Location occurs
municipality	The full, unabbreviated name of the next smaller administrative region than county (city, municipality etc.) in which the Location occurs. Do not use this term for a nearby named place that does not contain the actual location.
verbatimLocality	The original textual description of the place
habitat	A category or description of the habitat in which the Event occurred
decimalLatitude	The geographic latitude (in decimal degrees, using the spatial reference system given in geodeticDatum) of the geographic centre of a Location. Positive values are north of the Equator, negative values are south of it. Legal values lie between -90 and 90, inclusive
decimalLongitude	The geographic longitude (in decimal degrees, using the spatial reference system given in geodeticDatum) of the geographic centre of a Location. Positive values are east of the Greenwich Meridian, negative values are west of it. Legal values lie between -180 and 180, inclusive
geodeticDatum	The ellipsoid, geodetic datum or spatial reference system (SRS) upon which the geographic coordinates given in decimalLatitude and decimalLongitude as based [WGS 84]
coordinateUncertaintyInMeters	The horizontal distance (in metres) from the given decimalLatitude and decimalLongitude describing the smallest circle containing the whole of the Location. Leave the value empty if the uncertainty is unknown, cannot be estimated or is not applicable (because there are no coordinates). Zero is not a valid value for this term
verbatimElevation	The original description of the elevation (altitude, usually above sea level) of the Location
eventDate	The date-time or interval during which an Event occurred. For occurrences, this is the date-time when the event was recorded. Not suitable for a time in a geological context
year	The four-digit year in which the Event occurred, according to the Common Era Calendar
month	The integer month in which the Event occurred
day	The integer day of the month on which the Event occurred
recordedBy	A list (concatenated and separated) of names of people, groups or organisations responsible for recording the original Occurrence. The primary collector or observer, especially one who applies a personal identifier (recordNumber), should be listed first.
identifiedBy	A list (concatenated and separated) of names of people, groups or organisations who assigned the Taxon to the subject
datasetName	The name identifying the dataset from which the record was derived
language	A language of the resource [Russian]
license	A legal document giving official permission to do something with the resource [Creative Commons Attribution (CC-BY) 4.0 License]
references	A related resource that is referenced, cited or otherwise pointed to by the described resource

## Supplementary Material

FB3726CF-6CB3-5E0B-B9EC-527261AAD792 10.3897/BDJ.8.e59731.suppl1 Supplementary material 1 Vascular plants in the Herbarium of the Kandalaksha Strict Nature Reserve (KAND) Data type occurence File: oo_464278.txt https://binary.pensoft.net/file/464278 Mikhail N. Kozhin, Alexander N. Sennikov

## Figures and Tables

**Table 1.** Number of herbarium specimens collected in forest districts of the Kandalaksha Strict Nature Reserve and their adjacent areas

Territory	Number of specimens	Percentage
*Strictly protected areas*		
Barents Sea forest district	1746	17.09
Severnoe forest district	2613	25.56
Velikii Island forest district	2105	20.6
Terskoe forest district	2144	20.98
*Adjacent areas*		
Adjacent area of Barents Sea forest district	555	5.43
Adjacent area of Severnoe forest district	549	5.36
Adjacent area of Velikii Island forest district	142	1.39
Adjacent area of Terskoe forest district	365	3.57
**Total**	**10218**	**100**

**Table 2.** Areas of the Kandalaksha Strict Nature Reserve: characteristics and number of vascular plant specimens at KAND

**Area**	Strict protection date (second date denotes expansion)	Land size, ha	Number of islands	Number of specimens	Percentage
Ainovy Islands	1947	317	2	347	4.03
Gavrilov Archipelago	1969, 1991	95	14	151	1.75
Sem Ostrovov Archipelago and neighbouring territories	1938, 1947	3460	19	1248	14.5
Luvenga Archipelago	1967	413	42	535	6.22
Olenii Archipelago	1967	1452	72	672	7.81
Severnyi Archipelago	1932	1437	80	1315	15.28
Kibrinskii Archipelago	1967	71	5	90	1.05
Tarasikha Archipelago	1967	79	12	203	2.36
Vachev Archipelago	1967	185	5	49	0.57
Velikii Island and neighbouring territories	1940, 1967	11004	78	1549	18
Kem-Ludy Archipelago	1957	451	28	304	3.53
Porya Bay Islands	1967, 1977	1154	222	1808	21.01
Turii Mys	1977	829	0	336	3.9
**Total**	**1932–1991**	**20947**	**579**	**8607**	**100**
